# Diagnosis and Management of Acute Respiratory Distress Syndrome in a Time of COVID-19

**DOI:** 10.3390/diagnostics10121053

**Published:** 2020-12-06

**Authors:** Shayan Kassirian, Ravi Taneja, Sanjay Mehta

**Affiliations:** 1 Division of Critical Care Medicine, Centre for Critical Illness Research, Lawson Health Research Institute, London Health Sciences Center, London, ON N6A 5W9, Canada; skassirian2019@meds.uwo.ca (S.K.); ravi.taneja@lhsc.on.ca (R.T.); 2 Department of Medicine, Schulich Faculty of Medicine and Dentistry, Western University, London, ON N6A 5W9, Canada; 3 Department of Anesthesia and Perioperative Medicine, Schulich Faculty of Medicine and Dentistry, Western University, London, ON N6A 5W9, Canada; 4 Division of Respirology, Centre for Critical Illness Research, Lawson Health Research Institute, London Health Sciences Center, London, ON N6A 5W9, Canada

**Keywords:** acute respiratory distress syndrome (ARDS), COVID-19, SARS-CoV-2, high flow nasal oxygen, non-invasive ventilation, mechanical ventilation, dexamethasone

## Abstract

Acute respiratory distress syndrome (ARDS) remains a serious illness with significant morbidity and mortality, characterized by hypoxemic respiratory failure most commonly due to pneumonia, sepsis, and aspiration. Early and accurate diagnosis of ARDS depends upon clinical suspicion and chest imaging. Coronavirus disease 2019 (COVID-19) is an important novel cause of ARDS with a distinct time course, imaging and laboratory features from the time of SARS-CoV-2 infection to hypoxemic respiratory failure, which may allow diagnosis and management prior to or at earlier stages of ARDS. Treatment of ARDS remains largely supportive, and consists of incremental respiratory support (high flow nasal oxygen, non-invasive respiratory support, and invasive mechanical ventilation), and avoidance of iatrogenic complications, all of which improve clinical outcomes. COVID-19-associated ARDS is largely similar to other causes of ARDS with respect to pathology and respiratory physiology, and as such, COVID-19 patients with hypoxemic respiratory failure should typically be managed as other patients with ARDS. Non-invasive respiratory support may be beneficial in avoiding intubation in COVID-19 respiratory failure including mild ARDS, especially under conditions of resource constraints or to avoid overwhelming critical care resources. Compared to other causes of ARDS, medical therapies may improve outcomes in COVID-19-associated ARDS, such as dexamethasone and remdesivir. Future improved clinical outcomes in ARDS of all causes depends upon individual patient physiological and biological endotyping in order to improve accuracy and timeliness of diagnosis as well as optimal targeting of future therapies in the right patient at the right time in their disease.

## 1. Introduction 

Acute respiratory distress syndrome (ARDS) is a serious clinical illness, defined by severe hypoxemic respiratory failure, which continues to be associated with significant morbidity, mortality, and healthcare resource utilization. ARDS comprises 7–10% of admissions and 15–25% of mechanically ventilated patients in the intensive care unit (ICU), is fatal in 30–50% of patients, and costs on average over USD 90,000 per patient’s ICU stay [1,2,3,4,5].

ARDS has been intensively investigated for more than 50 years, resulting in our current understanding of a clinical-physiologic syndrome of lung inflammation and injury, biologically driven by a plethora of inflammatory cells and soluble molecules (i.e., cytokines). Despite greater understanding and multiple international clinical practice guidelines, ARDS remains under-recognized, the clinical importance is under-appreciated, and management is sub-optimal [2]. As such, many patients continue to suffer more severe, prolonged ARDS and worse clinical outcomes including higher mortality. Moreover, novel causes of ARDS, like coronavirus disease 2019 (COVID-19) are contributing to significant human disease and will undoubtedly continue to do so in the future. The global COVID-19 pandemic offers an important opportunity for all physicians to update their understanding of ARDS. 

Thus, we summarize current ideas on ARDS, including diagnosis, clinical/physiologic assessment and monitoring, as well as management. We will also highlight unique aspects of COVID-19 as an important, novel cause of ARDS. 

### 1.1. Causes of ARDS

**Figure 1:**
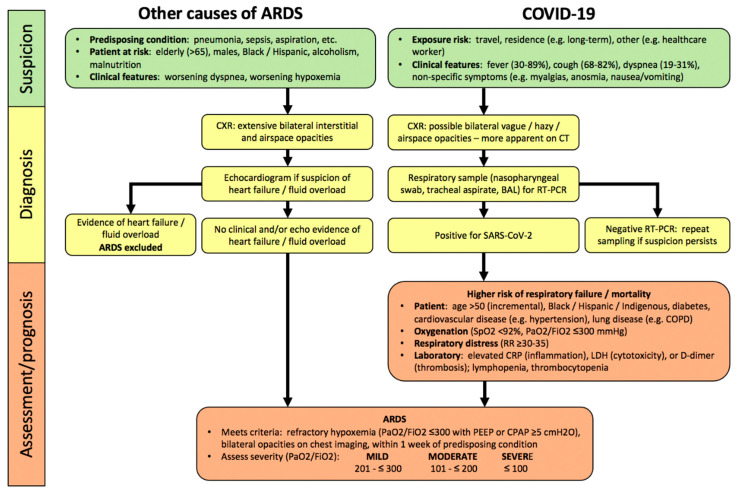
Algorithm for hospitalized patients at risk for acute respiratory distress syndrome (ARDS) and coronavirus disease 2019 (COVID-19). In contrast, to COVID-19, there are no specific lab abnormalities which adequately assess severity or predict prognosis in other causes of ARDS. Abbreviations: PaO2, partial pressure of oxygen in arterial blood; FiO2, inspired oxygen fraction; RR, respiratory rate; CRP, C-reactive protein; LDH, lactate dehydrogenase; PEEP, positive end-expiratory pressure; CPAP, continuous positive airway pressure.

The most common clinical conditions associated with development of ARDS include severe pneumonia (30–50%) and sepsis (25–30%; Figure 1), as confirmed in many large single- and multi-centred cohorts, including the Large Observational Study to Understand the Global Impact of Severe Acute Respiratory Failure (LUNGSAFE) registry, the largest cross-sectional study of ARDS patients admitted to intensive care units (ICUs) [2,6]. 

Pneumonia-associated ARDS is frequently due to bacterial infection (e.g., *Streptococcus pneumoniae, Staphylococcus aureus)*, but also develops with viral (e.g., influenza A) and fungal (e.g., *Pneumocystis jirovecii*) infections [6]. Coronavirus (CoV) causes of pneumonia and resulting ARDS have been recognized since the 2003 pandemic of severe acute respiratory syndrome (SARS). SARS-CoV-2 is a novel human coronavirus responsible for the pandemic known as COVID-19, first described in Wuhan, China in December 2019 [7]. Since then, more than 40 million COVID-19 cases globally have resulted in over 1 million deaths [8]. Although most infected individuals are asymptomatic or exhibit only mild symptoms, a significant minority of COVID-19 patients develop severe illness requiring hospitalization (10–14%), typically manifested as pneumonia [9,10]. 

Sepsis has long been recognized as a common and clinically important cause of ARDS. For example, sepsis was the primary cause of ARDS in 16% of cases in the LUNGSAFE registry and approximately 18% of patients with septic shock developed ARDS [2]. Moreover, sepsis-induced ARDS can have a worse prognosis than other causes of ARDS, typically because of the presence of co-morbid illnesses and higher risk of multiple organ dysfunction syndrome (MODS) [11,12].

### 1.2. Clinical Pathophysiology of ARDS

ARDS is characterized by the rapid development of severe lung inflammation causing damage to alveolar epithelial cells (AEC) and pulmonary microvascular endothelial cells (EC). Dysfunction of the alveolar-capillary endothelial barrier results in diffuse alveolar damage (DAD), which includes an initial exudative phase characterized by high-permeability, proteinaceous pulmonary interstitial and alveolar edema associated with the injury and death of EC as well as AEC desquamation, and a delayed fibroproliferative phase comprising fibrosis in intraluminal and interstitial compartments, and type II AEC proliferation (Table 1) [13,14]. Pathologic DAD is found in about half of patients with ARDS, and is associated with more severe hypoxemia and higher mortality [13].

Severe hypoxemia in ARDS is exacerbated by concomitant pathophysiologic disturbances, including surfactant dysfunction (reducing lung compliance causing atelectasis), pulmonary microvascular thrombosis (due to EC injury), higher physiological dead space, and increased shunt fraction due to impairment of hypoxic pulmonary vasoconstriction [15,16,17,18]. In addition, patients’ respiratory distress and strong inspiratory efforts can increase negative pleural pressure swings, increasing lung inflation stress, pulmonary blood flow and vascular pressures, potentially worsening pulmonary edema, collectively termed patient self-induced lung injury (P-SILI) [19]. 

The pathology of COVID-19-associated ARDS (COVID-ARDS) is also largely characterized by DAD, with some key differences such as lymphocyte rather than neutrophil predominance (Table 1). Some studies have highlighted more severe pulmonary microvascular EC injury associated with extensive microvascular thrombosis [20,21], for example, pulmonary vascular clot burden was up to nine times greater in COVID-ARDS versus influenza-associated ARDS [20]. However, such pulmonary microvascular findings have not been consistently observed in other pathologic descriptions [22,23,24]. The pathophysiology of respiratory failure in most patients with COVID-ARDS is also similar to other causes of ARDS, including atelectasis, low respiratory compliance, and intrapulmonary shunt [25,26]. It has been suggested that some COVID-ARDS patients manifest a different phenotype characterized by consolidation without atelectasis, preserved respiratory compliance, and more striking perfusion dysregulation, which may have treatment implications [27,28,29]. This remains an area of controversy and active clinical and physiologic research.

## 2. Diagnosis of ARDS

### 2.1. Clinical Assessment

By definition, ARDS develops within one week of onset or worsening of a predisposing condition (Figure 1), most commonly (>90%) within 48 h [1,2]. Demographic risks for developing ARDS are recognized (e.g., greater age, male sex, non-Caucasian ethnicity) [30,31]. ARDS diagnosis in hospitalized patients requires a clinical suspicion, based upon predisposing conditions, worsening oxygenation and dyspnea, bilateral interstitial and/or alveolar opacities consistent with pulmonary edema on chest radiograph (CXR), and exclusion of common causes of pulmonary edema (e.g., heart failure, fluid overload) clinically or with echocardiography [1]. 

A significant care-gap exists in the diagnosis of ARDS, especially mild ARDS which was unrecognized in 50% of patients in the large, global LUNGSAFE registry [2]. Indeed, mild ARDS is not a benign illness, as less than 20% of patients recovered within a week and overall in-hospital mortality was 29.7%. In addition, more than 40% of mild ARDS progressed to moderate–severe ARDS which was associated with higher mortality of 35–42.9% [32].

Most COVID-19 patients develop symptoms of fever, cough, and dyspnea within 5 days of infection (Figure 1). Hospitalized patients can deteriorate quite rapidly within hours to a few days, manifesting worsening hypoxemia and respiratory distress as features of severe pneumonia, and 20–30% develop COVID-ARDS [9,10,33,34]. Compared to patients with other causes of ARDS, pulmonary opacities are less obvious on CXR (54–76%) in COVID-ARDS patients [35,36]. Chest CT scan is clearly more sensitive to the presence of abnormalities in patients with confirmed COVID-19, with a sensitivity of 93.1% (95% CI: 90.2–95.0) in a meta-analysis (65 studies; 5759 patients) [37], but abnormalities are poorly specific for a diagnosis of COVID-19 compared to other respiratory infections. 

### 2.2. Assessment of Severity

ARDS severity is assessed by the degree of hypoxemia, quantified by the ratio of arterial partial pressure of oxygen (PaO_2_) to the fraction of inspired oxygen (FiO_2_) as per the Berlin criteria, which is strongly predictive of worsening survival (Figure 1) [1]. In addition, the presence of hypercapnia (PaCO_2_ ≥ 50 mmHg) was independently associated with more organ dysfunction and higher mortality [38]. Other laboratory abnormalities have not been shown to assess severity or predict prognosis in ARDS, but key investigations can identify prognostically-important complications of non-pulmonary organ dysfunction, e.g., cardiac, renal, and potentially MODS [39]. 

In patients with COVID-19, severity of pneumonia and respiratory failure is also assessed by the degree of hypoxemia, including arterial oxygen saturation by pulse oximetry (SpO2), and the PaO_2_/FiO_2_ ratio [26,40]. COVID-19 is associated with distinct laboratory abnormalities which predict greater risk of respiratory failure and worse clinical outcomes including higher mortality, independent of the severity of ARDS. These include markers of inflammation (elevated C-reactive protein (CRP)), cytotoxicity (increased lactate dehydrogenase (LDH)), and both macrovascular and microvascular thrombosis in systemic and pulmonary circulations (higher D-dimer levels), as well as lymphopenia (Figure 1) [41,42]. It has been suggested that these markers should be assessed at baseline in hospitalized COVID-19 patients [40,43], however the clinical utility of serial monitoring has not yet been established.

Given the presence of multiple physiologic and laboratory abnormalities in COVID-19, there may be more robust prognostic value in assessing a combination of parameters. For example, in a multicentre, observational retrospective study of patients being assessed in the ED, a model developed through machine-learning, the Quick COVID-19 Severity Index comprising three respiratory parameters (FiO_2_, SpO_2_, respiratory rate (RR)) was predictive of the risk of respiratory failure within the first 24 h of admission [44]. Following hospital admission, another machine-learning composite score, which included age, lymphocyte count and levels of inflammatory markers (e.g., LDH, CRP), was found to best predict the risk of severe hypoxemic respiratory failure, need for ICU admission and/or invasive respiratory support, and mortality in hospitalized COVID-19 patients [45]. Finally, in COVID-19 patients with ARDS, a multicentre, observational study identified the highest risk of mortality was associated with both reduced respiratory compliance and higher D-dimer levels [46]. 

## 3. Management of Patients with ARDS

### 3.1. General Approach

Management of ARDS remains largely supportive, including treatment of the predisposing condition, as there are no specific medical therapies that address the lung inflammation and alveolo-capillary injury. Standard care for ICU-admitted patients includes early nutritional support, appropriate analgesia, sedation, thromboprophylaxis, semi-recumbent position, gastric ulcer prophylaxis, and glycaemic control (FASTHUG) [47]. In ARDS patients, the frequent presence of non-pulmonary organ dysfunction or development of MODS contributes to severity of illness, intensity of required care, and mortality [12,48]. Similarly, thirty-to-fifty percent of critically-ill COVID-19 patients will develop non-pulmonary organ dysfunction leading to MODS, which is the most common cause of mortality [34,36,49]. 

Many respiratory support modalities are high-risk aerosol-generating medical procedures requiring specific attention, during the care of COVID-19 patients, to minimization of unnecessary staff exposure, appropriate contact precautions, and airway management expertise. Physicians are encouraged to follow local guidelines for safe application and monitoring of all respiratory support and associated procedures, e.g., high-flow nasal-cannula O_2_ (HFNO), non-invasive positive pressure ventilation (NIPPV), intubation, mechanical ventilation (MV), bronchoscopy [40]. 

### 3.2. Respiratory Support of Mild ARDS 

Initial respiratory support of patients with hypoxemia consists of supplemental O_2_ [50]. Specific SpO_2_ targets in various patient populations remain uncertain, given competing goals of addressing persistent hypoxemia as well as avoiding hyperoxia, both of which may be associated with increased mortality [51,52]. In ARDS, permissive hypoxemia is not recommended [53,54,55,56]. For example, conservative O_2_ (SpO_2_ 88–92%) was associated with a non-significant higher risk of 28-day mortality, but higher 90-day mortality and more intestinal ischemia than more liberal O_2_ (SpO_2_ ≥ 96%) [53]. In persistent hypoxemic respiratory failure despite maximal supplemental O_2_ by facemask, various non-invasive respiratory support modalities may be considered, and clearly are being commonly employed recently in COVID-19 patients [57,58,59]. 

#### 3.2.1. High-Flow Nasal-Cannula O_2_ (HFNO)

**Figure 2:**
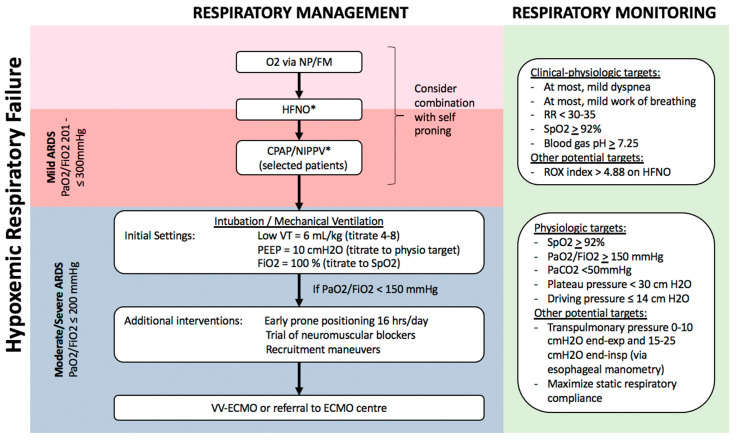
Algorithm for respiratory management of patients with hypoxemic respiratory failure. Patients with hypoxemia despite supplemental O_2_, including those who meet criteria for mild ARDS, can potentially be managed with non-invasive respiratory support, including HFNO and NIPPV, possibly in combination with self-proning. Sequential escalation of non-invasive respiratory support modalities should be considered unless clinical-physiologic targets are met, depending on local critical care expertise and resources. Patients with moderate–severe ARDS require invasive mechanical ventilation with monitoring and adjustment of ventilatory parameters to minimize ventilator-induced lung injury (VILI), and may benefit from additional measures to improve oxygenation such as prone positioning, recruitment manoeuvres, and potentially veno-venous extra-corporeal membrane oxygenation (VV-ECMO). Notes: a. Non-invasive respiratory support with HFNO/CPAP/NIPPV requires careful monitoring for lack of improvement or persistent respiratory distress, and consideration of intubation/mechanical ventilation. b. All non-invasive and invasive respiratory support modalities are high-risk aerosol-generating medical procedures which should be carried out by experts in airway management, with appropriate precautions (e.g., minimal staff in room, N95, negative pressure room). c. Tidal volume is referenced to predicted body weight. d. Recruitment manoeuvres requires sustained inflation, e.g., inspiratory hold at 35–40 cm H_2_O for set time (e.g., 40 s). Stepwise recruitment (with incremental levels of PEEP) is not recommended. e. ROX index = SpO2/FiO2/Respiratory Rate. f. Plateau pressure = airway pressure after 0.5 s pause at end-inspiration. g. Driving pressure = plateau pressure—PEEP. h. Transpulmonary pressure = airway pressure—pleural pressure (under zero flow conditions). i. Static respiratory compliance = tidal volume/(plateau pressure—PEEP). Abbreviations: NP, nasal prongs; FM, facemask; HFNO, high-flow nasal oxygen; CPAP, continuous positive airway pressure; NIPPV, non-invasive positive pressure ventilation; RR, respiratory rate; VT, tidal volume; PEEP, positive end-expiratory pressure; ECMO, extra-corporeal membrane oxygenation. * These interventions, while increasingly being used globally, especially during the COVID-19 pandemic, are not yet supported by robust evidence in patients with ARDS.

This is a novel technique which can improve oxygenation in hypoxemic respiratory failure (Figure 2), through several mechanisms including higher inspired O_2_ concentrations ≤90%, decreased dead space, and increased lung volume through generation of a low-level of continuous positive airway pressure (CPAP) [60,61]. In the largest randomized controlled trial (RCT) of HFNO vs. standard O_2_ therapy in patients with hypoxemic respiratory failure (the absence of use of CPAP meant that ARDS could not be formally diagnosed based on Berlin criteria), HFNO reduced 90-day mortality by 50% but there was no difference in the need for invasive respiratory support through intubation/MV [60]. A retrospective review and two meta-analyses have concluded that HFNO was associated with 15–24% reduced risk of subsequent intubation in hypoxemic respiratory failure, but did not reduce duration of hospital or ICU admission or improve survival [62,63,64]. 

HFNO has more commonly been used in the management of hypoxemic respiratory failure in COVID-19 patients, depending on geography and access to other respiratory support measures [57,65,66]. For example, 5–64% of moderate–severe hypoxemic COVID-19 patients in Italy, China, and the US were initially supported with HFNO [42,67,68,69]. In a retrospective review of the largest single-centre series of 104 COVID-19 patients with moderate–severe hypoxemia, 64% of those treated with HFNO avoided intubation and had mortality of 2.9%, compared to 14.4% in those requiring subsequent intubation/MV [63].

#### 3.2.2. Continuous Positive Airway Pressure (CPAP)/Non-Invasive Positive Pressure Ventilation (NIPPV)

In patients with persistent hypoxemia despite maximal supplemental O_2_ by either facemask or HFNO, a trial of either CPAP (via nasal/facemask or hood/helmet) or NIPPV via facemask can be considered. CPAP/NIPPV may be beneficial in improving oxygenation and respiratory distress, decreasing FiO_2_ requirements, and possibly reducing the need for invasive support through intubation/MV [70,71]. For example, the above-cited network meta-analysis of non-invasive respiratory support in hypoxemic respiratory failure reported that both helmet NIPPV (RR 0.26, 95%CI 0.14–0.46) and facemask NIPPV (RR 0.76, 95%CI 0.62–0.90) reduced the risk of subsequent intubation and were both associated with reduced risk of death compared to supplemental O_2_ [62,72]. 

CPAP/NIPPV is commonly being used globally for patients with hypoxemic respiratory failure including ARDS, e.g., 15.5% of ARDS patients in the global LUNGSAFE registry [70]. However, there is a clear risk of failure of such non-invasive respiratory support, as 22.2% of mild and 42–47% of moderate–severe ARDS patients failed CPAP/NIPPV trial within 2 days, experiencing lack of improvement or worsening of respiratory distress and/or hypoxemia [70]. During NIPPV trials, careful respiratory monitoring is essential because clinical outcomes are worse in patients who fail NIPPV, possibly because of delayed definitive management of respiratory failure with intubation and MV [73,74]. For example, patients with hypoxemic respiratory failure who failed NIPPV had longer ICU and hospital stay, as well as more than four-fold higher mortality [74]. Thus, NIPPV may be beneficial in patients with mild ARDS (Figure 2), but this specific respiratory support measure has not been specifically recommended in recent guidelines [48,75]. 

Non-invasive respiratory support with CPAP/NIPPV is also being increasingly instituted in COVID-19 patients, especially under local conditions of constrained ICU resources [59]. For example, 3–56% of hypoxemic COVID-19 were treated with CPAP/NIPPV, with higher rates of usage in critically ill and moderate–severe patients [7,10,33,49,66,67,68,69,76,77]. Several uncontrolled reports suggested a reduced need for intubation, but only a single controlled study has addressed this, using a retrospective, historical time period-controlled cohort design, reporting significantly higher intubation-free survival at 7 days with CPAP [78]. While such non-invasive respiratory support measures may be appropriate in some COVID-19 patients with hypoxemic respiratory failure, specifically those who either do not yet meet criteria for ARDS or have mild ARDS (Figure 2), current guidelines do not provide any specific recommendations in the absence of more robust data [40,43,59,79]. 

#### 3.2.3. Prone Positioning

Based on strong evidence for improved clinical outcomes in ARDS patients who are intubated and ventilated (see Section 3.3. Respiratory Support of Moderate–Severe ARDS below [80]), prone positioning is being increasingly used to improve oxygenation in spontaneously-breathing non-intubated patients with hypoxemic respiratory failure, including COVID-19. For example, in a small prospective cohort study of 20 patients with ARDS, prone positioning combined with either HFNO or NIPPV was associated with reduced need for intubation/MV only in patients with moderate ARDS, not in those with severe ARDS [81]. 

Several uncontrolled series have reported that self-proning may improve oxygenation in spontaneously breathing COVID-19 patients receiving supplemental O_2_ or other non-invasive respiratory support (e.g., HFNO, CPAP/NIPPV) [65,82,83,84,85]. In the first reported series of 50 COVID-19 patients managed in the emergency department, oxygenation improved from an average of 84% on supplemental O_2_ to 94% after self-proning for 5 min [82]. In addition, 64% of patients with unspecified repeated self-proning sessions recovered to hospital discharge without intubation/MV. Self-proning was not tolerated, including worsening oxygenation and/or respiratory distress, in 13–25% of patients [84,86,87]. Moreover, although oxygenation improves in most patients when prone, the improvement is maintained in only about 50% of patients when resuming the supine position, with some evidence that proning may be more effective earlier in the hospital course and specifically in patients with higher inflammatory markers (e.g., CRP, LDH) [84]. Early oxygenation improvement has been associated with reduced need for subsequent intubation/MV in some studies [87,88] but not in others [84]. In summary, self-proning is currently widely employed in the management of COVID-19 patients globally in the absence of strong evidence of improved outcomes and there are no clear recommendations regarding specifics of patient selection, duration and frequency of proning sessions. Self-proning is not feasible or tolerable for all patients, is associated with clear risks, including inadequate respiratory support in patients with respiratory distress and/or high work of breathing which are associated with higher risk of P-SILI and progressive lung injury [19,89,90]. As such, prone positioning in spontaneously breathing patients mandates rigorous clinical and respiratory monitoring for lack of improvement and/or persistent respiratory distress in order to facilitate timely intubation/MV. 

### 3.3. Respiratory Support of Moderate–Severe ARDS

In moderate–severe ARDS patients, respiratory management is similar for ARDS from all causes including COVID-19 (Figure 2) [26,40,48,75,79,91]. Invasive respiratory support through endotracheal intubation and MV is strongly recommended for worsening or persistent respiratory distress, persistent hypoxemia (SpO_2_ < 92%), or progressive hypercapnia. In patients requiring MV, specific ventilatory modalities and parameters are guidelines-recommended based on improved outcomes in multiple RCTs (Figure 2) [48,75,91]. The goal is to use a lung protective strategy to prevent excessive lung tidal-inflation stress (volutrauma) and cyclic atelectasis-recruitment (atelectrauma), reducing the risk of ventilation-induced lung injury (VILI) [92]. The most important measure is MV using low-tidal volumes, specifically a target of 4–8 mL/kg predicted body weight [91,93]. 

The application of positive end-expiratory pressure (PEEP) is essential in order to reduce atelectasis and maximize respiratory compliance, and PEEP is optimally selected to avoid excessive plateau and driving pressures (Figure 2) [91,93,94]. Novel physiologic monitoring using oesophageal manometry may allow optimization of PEEP in individual patients, although the benefit of such an approach in terms of clinical outcomes remains uncertain [95,96]. Additionally, early prone positioning should be implemented as a lung protective measure, as it has been shown to reduce 28-day mortality by 16% when implemented 12–24 h after initiation of MV [80].

Several weak recommendations suggest approaches for management of persistent hypoxemia, patient-ventilator dyssynchrony, or low lung compliance with high plateau or driving pressures (Figure 2). These include short courses of neuromuscular blockade-induced paralysis, and specific recruitment manoeuvres [91,97,98]. Refractory hypoxemia not responding to conventional therapy warrants consideration of veno-venous extra-corporeal membrane oxygenation (VV-ECMO). Besides directly improving hypoxemia and related multiple organ dysfunction, ECMO may offer more homogeneous, ultraprotective ventilation. In brief, ECMO should be considered when patients have (a) persistent PaO_2_/FiO_2_ <50 mmHg for >3 h or <80 mmHg for >6 h despite FiO_2_ > 80% and PEEP >10, or (b) pH < 7.25 with PaCO2 > 60 mmHg for >6 h. If ECMO is not available locally, patients with severe respiratory failure should be considered for transfer to a high-volume facility with ECMO expertise, if clinically feasible. VV-ECMO achieves similar outcomes in all causes of ARDS, including COVID-ARDS [99,100]. 

### 3.4. Medical Approaches to ARDS Therapy

Given the central contribution of alveolo-capillary injury and high-permeability pulmonary edema to refractory hypoxemia in ARDS, conservative fluid management after initial resuscitation may reduce edema, improve gas-exchange, and improve clinical outcomes such as decreased duration of MV and ICU length of stay (Table 2) [91,101]. Regardless of the primary cause of ARDS, the presence of concomitant bacterial infection should be investigated, and broad-spectrum antibiotic therapy considered. Limited evidence indicates early systemic steroids may reduce duration of MV and mortality, but there are conflicting recommendations regarding dose, timing, and consideration in individual patients (Table 2) [91,102]. A multitude of RCTs of various anti-inflammatory and pathophysiology-based therapies have failed to improve clinical outcomes, such that there is no specific medical therapy currently indicated or recommended for lung inflammation and injury in ARDS patients. 

There is active research into various anti-viral and anti-inflammatory therapies specifically for SARS-CoV-2 infection resulting in COVID-19 pneumonia and/or ARDS (Table 2) [79,103]. Strong evidence supports that corticosteroids (i.e., dexamethasone) reduce the need for ICU admission and intubation/MV in hospitalized, hypoxemic COVID-19 patients. Moreover, in COVID-ARDS patients, corticosteroids shorten the duration of MV and reduce mortality [104]. As such, corticosteroids are strongly recommended for hypoxemic COVID-19 patients [104,105]. Remdesivir is the first antiviral drug found to have some clinical benefit, namely in reducing time to recovery [106]. Many putative therapies are in ongoing clinical trials with some promise of preventing or treating COVID-19, including human convalescent plasma, systemic anticoagulation, and 25-hydroxy vitamin D [107,108,109]. A number of other medical therapies have been considered but have shown no benefit, including lopanivir/ritonavir and hydroxychloroquine [110,111]. There is concern around the routine clinical use of unproven experimental therapies, including high risk of drug–drug interactions given that the majority of hospitalized COVID-19 patients are older with multiple co-morbidities requiring treatment with many other medications [112].

## 4. Outcomes in Patients with ARDS

Mortality in ARDS has clearly improved from initial reports of 70–80% but still remains about 40% [2]. Although obstructive and restrictive pulmonary function defects are commonly found in survivors, survivors are infrequently limited by respiratory issues but most commonly suffer from long-term physical disabilities as well as cognitive and psychological issues [113,114]. Significant pulmonary fibrosis in a minority of patients may contribute to poorer outcomes [115]. 

The vast majority of COVID-19 patients are admitted to ICU because of respiratory failure (80–90%), with most requiring MV (60–80%) and experiencing significant but widely varying mortality (26–90%) [9,33,49]. There is early concern about potential pulmonary fibrosis in patients with COVID-19 pneumonia, as well as poorly understood long-term constitutional, pulmonary, and systemic symptoms sometimes labelled “chronic COVID”. Moreover, long-term cognitive and physical disabilities in critically ill COVID-19 patients, similar to other ARDS patients, are expected given the duration of MV and ICU care [116]. More robust mortality and long-term outcomes await further research. 

## 5. Future Management of Patients with ARDS

The aforementioned multitude of negative pharmacologic RCTs in ARDS has resulted in a lack of available effective medical therapies. This is due, to a large extent, to the simplification of previous and current ARDS clinical-physiologic definitions which do not adequately reflect the heterogeneity of ARDS populations, characterized by inter-individual variation in cause, severity, respiratory physiology, and outcomes, as well as broad ranges of patient demographics (e.g., age, race, gender) and comorbidities. Even more important, biological differences between patients driven by genetics, genomics, and environmental influences collectively determine individual patient-specific complex physiologic, immune, inflammatory and cell-injury responses, termed patient “endotype”. The concept of precision medicine endeavours to identify and use such biological variation in individuals in order to improve diagnosis, as well as target treatment to smaller, focused groups of patients who are more likely to experience benefit and/or less likely to suffer adverse effects from targeted therapies. 

There is evidence in patients with ARDS that such endotypes strongly determine the variable responses of individuals to treatment, including MV parameters, fluid strategy, and pharmacologic therapies [117,118,119]. For example, despite a lack of overall benefit of simvastatin in ARDS, a post-hoc analysis identified a mortality benefit of simvastatin in patients with a hyperinflammatory endotype characterized by higher levels of pro-inflammatory mediators (e.g., IL-6, IL-8, s-TNFr1), metabolic acidosis, and higher vasopressor requirements [117]. As such, specific endotypes based most likely on a combination of validated clinical (e.g., demographics, ARDS severity) and biologic variables (e.g., genetic polymorphisms, levels of cytokines such as IL-6), may best identify prognosis of individual patients (prognostic enrichment) as well as optimal responses to and tolerability of novel therapies (predictive enrichment) [120]. A precision-medicine approach is already being utilized in order to reduce clinical trial heterogeneity by recruitment of more homogeneous populations or stratification of subjects based on patients’ endotypes. 

In COVID-ARDS, clinical and biological phenotypes likely determine outcomes as well as response to therapies, for example, low and high lung-compliance phenotypes [27,28]. Combinations of clinical features and laboratory abnormalities (Figure 1) reflective of COVID-19 severity could identify specific phenotypes with prognostic or therapeutic relevance [41]. For example, more severe respiratory failure and a high D-dimer may indicate greater pulmonary vascular injury and thrombosis; as such, systemic anticoagulation could improve outcomes in patients with such a “hypercoagulable” phenotype [121]. Clearly, active current research will better define COVID-19 endotypes and establish endotype-specific diagnostic and/or management approaches. 

## 6. Conclusions

ARDS remains a common and serious illness which is expected to have ongoing significant morbidity, mortality and healthcare resource impacts, given recent and likely future new causes, such as SARS-CoV-2. Strong basic science and clinical research continue to define the biology of ARDS, identify most effective and safe management practices, including currently largely supportive measures such as intubation and MV as well as novel respiratory approaches (e.g., HFNO, NIPPV), and multiple international guidelines summarize evidence-based recommendations. A significant care gap remains around targeted medical therapies, but precision medicine approaches to RCTs and clinical management hold promise for improved clinical management and patient-relevant outcomes in patients with ARDS, including in COVID-19.

## Figures and Tables

**Table 1:** Pulmonary pathology features of COVID-19-associated ARDS (COVID-ARDS) versus other causes of acute respiratory distress syndrome (ARDS).

Pathology	ARDS	COVID-ARDS
**Diffuse Alveolar** **Damage (DAD)**	***Early/Exudative:*** - interstitial/alveolar edema - “hyaline” membranes - neutrophil infiltration - AEC desquamation - pulmonary microvascular thrombosis ***Late/Fibroproliferative:*** - alveolar/interstitial fibrosis - type II AEC hyperplasia	***Similar to ARDS except:*** - paucity of neutrophils - interstitial/alveolar lymphocytic infiltration - possibly increased pulmonary microvascular thrombi relative to other causes
**Other features**	- organizing pneumonia (fibrosis) - alveolar haemorrhage - viral pneumonia	- occasional viral cytopathic changes (multinucleated syncytial cells, atypical enlarged AEC) - viral inclusions in AEC

Abbreviations: AEC, alveolar epithelial cells.

**Table 2:** Medical treatment approaches for ARDS and specifically for COVID-19-associated ARDS.

Intervention	ARDS	COVID-ARDS
Fluid management		
Conservative fluid strategy	Weak recommendation post initial resuscitation (SCCM [48], FICM-ICS [75])	Weak recommendation (SSC [40])
Anti-inflammatory therapy		
Steroid	Weak recommendation - Methylprednisolone 1–2 mg/kg/d with 14 d taper (FICM-ICS [75], SCCM-ESICM [102]	Recommended - Dexamethasone 6 mg/d for 10 d (WHO [33], IDSA [79], CMAJ [103])
Other (Physiologic/Biologic)	Not recommended - β2-agonists - Exogenous surfactant - Anti-IL1β - Statins	Not recommended - Hydroxychloroquine/chloroquine - Lopanivir/ritonavir
Experimental	Current trials - Anti-tissue factor antibody fragment - MAPK inhibitor - Stem cell therapies - Complement inhibitor - JAK inhibitor	Current trials- Convalescent human plasma - Intravenous Immunoglobulin - IL-6 inhibitor (e.g., tocilizumab) - IL-1 inhibitor (e.g., anakinra) - Anti-GM-CSF (e.g., mavrilimumab) - Anticoagulants (e.g., Low molecular weight heparin) - Fibrinolytics (e.g., tPA) - 25-OH vitamin D
Anti-microbials		
Antibiotics	Strong recommendation - If ARDS due to pneumonia or sepsis (SCCM [48]) - If evidence of ventilator-associated pneumonia (SCCM [48])	Weak recommendation - In patients requiring MV (SSC [40], IDSA [79]) - If concomitant bacterial pneumonia (SSC [40], IDSA [79]))
Antivirals	Specific viral targeted therapy indicated - If viral infection identified (e.g., influenza, RSV)	Specific viral targeted therapy indicated - If evidence of concomitant viral pneumonia (e.g., influenza, RSV) SARS-CoV-2 targeted therapy - Remdesivir (IDSA [79])
